# Development of Quantitative and Temporal Scalar Implicatures in a Felicity Judgment Task

**DOI:** 10.3389/fpsyg.2018.02763

**Published:** 2019-02-18

**Authors:** Walter Schaeken, Bojoura Schouten, Kristien Dieussaert

**Affiliations:** ^1^Laboratory of Experimental Psychology, KU Leuven, Leuven, Belgium; ^2^Health Care, Faculty of Medicine and Life Sciences, Hasselt University, Hasselt, Belgium; ^3^Quality Care, UC Leuven-Limburg, Leuven, Belgium

**Keywords:** pragmatics, experimental pragmatics, scalar implicature, Felicity Judgment Task, informational strength, alternatives

## Abstract

Experimental investigations into children’s interpretation of scalar terms show that children have difficulties with scalar implicatures in tasks. In contrast with adults, they are for instance not able to derive the pragmatic interpretation that “*some*” means “*not all*” (Noveck, 2001; Papafragou and Musolino, 2003). However, there is also substantial experimental evidence that children are not incapable of drawing scalar inferences and that they are aware of the pragmatic potential of scalar expressions. In these kinds of studies, the prime interest is to discover what conditions facilitate implicature production for children. One of the factors that seem to be difficult for children is the generation of the scalar alternative. In a Felicity Judgment Task (FJT) the alternative is given. Participants are presented with a pair of utterances and asked to choose the most felicitous description. In such a task, even 5-year-old children are reported to show a very good performance. Our study wants to build on this tradition, by using a FJT where not only “*some-all*” choices are given, but also “*some-many*” and “*many-all.*” In combination with a manipulation of the number of successes/failures in the stories, this enabled us to construct control, critical and ambiguous items. We compared the performance of 59 5-year-old children with that of 34 11-year-old children. The results indicated that performance of both age groups was clearly above chance, replicating previous findings. However, for the 5-year-old children, the critical and ambiguous items were more difficult than the control items and they also performed worse on these two types of items than the 11-year-old children. Interestingly with respect to the issue of scalar diversity, the 11-year-old children were also presented temporal items, which turned out to be more difficult than the quantitative ones.

## Introduction

Consider a brainstorm session for some new research lines, where the head of the research group offers the following feedback: “*Some of John’s ideas were interesting*." The use of “*some*” seems to lead to the inference that the speaker did not find all of John’s ideas interesting. Different theories try to explain this kind of inferences. “*Some*” seems to invoke “*all*,” which is the more informative. Therefore, “*some*” is strengthened by the negation of “*all.*” The latter step can be made on the basis of pragmatic reasoning or can be based on grammar.

In Grice’s terms (Grice, 1975), the explanation goes as follows. Given the cooperation principle guiding communication (“Make your contribution such as it is required, at the stage at which it occurs, by the accepted purpose or direction of the talk exchange in which you are engaged”), one should try to say no more and no less than is required for the purpose of the exchange (the Quantity-maxim). Therefore, the head of the research group who said that *some* of the ideas were interesting does not think that the alternative and more informative *all*-sentence is true. Moreover, if the addressee assumes that the head of the research group has an opinion about the truth of the *all*-sentence (see Sauerland, 2004 for a definition of the Opinionated Speaker; see also Fox, 2007), the addressee will conclude that the head believes that the *all*-sentence is false and that, therefore, the head thinks that not all of John’s ideas were interesting. It is important to note that from a logical point of view one can use “*some*” when “*all*” is the case. Indeed, the lower-bounded semantics of “*some*” is “*at least some and possibly all*” (Horn, 1972). The scalar implicature (SI) corresponds to the upper-bounded meaning (“*but not all*”) and can be seen as a pragmatic enrichment of the semantic content of the quantifier. Hence, in a situation where the assertion “*all of John’s ideas*” is true, the *some*-sentence is acceptable according to the semantic, lower-bounded interpretation of the scalar term, but unacceptable according to its pragmatic, upper-bounded interpretation. As said before, grammatical accounts (e.g., Chierchia, 2004; Fox, 2007) share some basic aspects with a Gricean account, but are clearly different in their assumption that underinformative sentences are ambiguous between different syntactic structures. In grammatical accounts, a covert syntactic operator is introduced, whose meaning is close to “*only.*” Of the possible alternatives, the operator excludes all those that are more informative as the proposition expressed by the sentence without the operator (Geurts, 2010). In our example, appending the operator leads to the proposition that the head of the research group liked some of the ideas and the negation of the proposition that she liked all of them, which can be paraphrased into “*she thinks that only some of the ideas were interesting.*”

Experimental research has been devoted to the interpretation of scalars, with a strong focus on “*all-some*,” probably because this scale offers a sharply defined and easily testable division between the encoded and the inferred meaning. When adults are presented with problems like the one above (“*some of the ideas were interesting*”), they overwhelmingly chose the pragmatic interpretation, that is, the inference from “*some*” to “*not all*” (e.g., Noveck, 2001; Bott and Noveck, 2004; De Neys and Schaeken, 2007; Marty and Chemla, 2013; Heyman and Schaeken, 2015; van Tiel and Schaeken, 2017). On classical tasks, like the Truth Value Judgment Task (TVJT) where one has to indicate whether an utterance if true or false, young children perform poorer than adults in deriving these SIs. They more often prefer the logical answer; hence they accept underinformative scalar sentences (see e.g., Chierchia et al., 2001; Noveck, 2001; Papafragou and Musolino, 2003; Foppolo et al., 2012; Janssens et al., 2015).

However, these findings do not mean that young children are unable to show more adult-like behavior when interpreting scalar statements. Several factors seem to be able to lift the performance of young children (for an overview and a nice series of experiments, see also Foppolo et al., 2012). One of the factors is awareness of the goal and training, as demonstrated by Papafragou and Musolino (2003). Before the start of the experiment, the researchers caused an enhanced awareness of the goals of the task and gave a short training to detect infelicitous statements. As a result, children’s sensitivity to SI significantly improved, although they still fell short of a fully mature performance. Another factor is the nature of the task. Pouscoulous et al. (2007) did not ask for a truth evaluation, but asked children to perform an action. In order to realize this, they presented the children with five boxes and five tokens. Pouscoulous and her colleagues requested children to adapt the boxes to make them compatible with a statement. For example, the children saw that all five boxes contained a token and were told ‘*I would like some boxes to contain a token.*’ Pouscoulous et al. (2007) reasoned that if the children believed that “*some”* is compatible with “*all*,” they should leave the boxes unaltered; otherwise they should remove at least one token. The results showed that the number of derived implicatures in children increased. The nature of the answer is also an important factor. Katsos and Bishop (2011) focused on the fact that underinformative statements are true but suboptimal: in a binary judgment task, one cannot express being aware of the suboptimality. Indeed, one is forced to choose between “*true*” and “*false.*” If one is tolerant to this suboptimality and focuses more on the fact that these statements are logically correct, one goes for “*true.*” Katsos and Bishop (2011) offered a third response option (corresponding to “*both true and false*”) and observed that both adults and young children went overwhelmingly for this middle option, thereby showing sensitivity for informativeness.

The research sketched above shows that the failure observed in classic TVJT-tasks does not reflect a genuine inability to derive SIs. This motivated us to move away from this demanding classic task and to use another task in our experiments, that is, a Felicity Judgment Task (FJT; see e.g., Chierchia et al., 2001). In this task, participants are presented with two alternative descriptions of the same situation and they have to decide which one is the best. One advantage of this task is that the scalar alternatives are explicitly presented, and therefore participants do not have to generate them.

Indeed, one factor that recently received attention is the cognitive availability of the scalar alternatives, that is, the ability to generate the relevant alternative that is going to be used to undergo the SI-process. Consider the task of a pre-schooler who observes a situation where three mice enter a hole. Next the child is asked to evaluate a sentence like “*some of the mice entered the hole.*” The pragmatic response “*no, that’s not a good sentence*” requires them to generate the stronger alternative (“*all of the mice entered the hole*”) and compare the information strength. Interesting in this respect is a study by Barner et al. (2011). Four-year-old children were for instance presented with a situation where Cookie Monster was holding three pieces of fruit (and no other pieces of fruit were available in the context). When they were asked whether Cooking Monster was holding only some of the food, the majority said “*yes.*” When asked whether Cookie Monster was holding only the banana and the apple, they overwhelmingly said “*no.*” Hence, when the alternatives were provided contextually, as in the last question, children were able to assign strengthened interpretations to utterances when these included the focus element “*only.*” For the context-independent scale *some/all*, children were not able to do this. In a sentence-picture verification task, Skordos and Papafragou (2016) manipulated the accessibility of the alternative by varying the order of trials. They compared performance of 5-year-old children in the condition in which the trials with “*some*” were presented before the trials with “*all*” with the mixed condition (in which trials with “*some*” and “*all*” were intermixed in a pseudorandom order). In the latter condition, children derived more SIs, probably due to the fact that alternatives were more accessible. In two follow-up experiments, Skordos and Papafragou (2016) showed the importance of relevance. Children used the explicitly mentioned stronger alternative for SI-generation only when the alternative was relevant. In two experiments, with a modified TVJT, Tieu et al. (2016) showed that as early as 4 years old, children can compute free choice inferences. However, they were not able to compute SIs. As an explanation, they offered the restricted alternatives hypothesis: Children have the ability to compute inferences arising from alternatives whose construction does not require access to the lexicon. Because the alternatives from which free choice inferences arise are contained within the assertion, they can be computed. The alternatives of SIs are typically not contained within the assertions and therefore these implicatures are hard. Tieu et al. (2016) also state explicitly that mentioning alternatives helps children to compute the corresponding inferences.^1^

In the current study we use an adapted version of the FJT to investigate further the role of alternatives. Chierchia et al. (2001) investigated if, on their way to full mastery of scalar terms, children might pass through a stage in which they know already some aspects of them. More specifically, Chierchia et al. (2001) examined situations where the children knew that “*and*” truly applies, and tested if children prefer “*and*” above “*or*” through a FJT. Fifteen 5-year-old children were presented with two alternative descriptions of the same situation and they had to decide which one was the best. Remarkably, with the presence of the relevant alternative representations, the children consistently applied SIs. It has to be emphasized that this task does not require the actual derivation of SIs: Comparing the informativity of the competing utterances and applying the Maxim of Quantity will lead to the appropriate response. Foppolo et al. (2012) presented a rather small set of 17 5-year-old children with a similar task, now employing the terms “*some*” and “*all.*” In line with Chierchia et al. (2001), the children’s performance in this FJT was above 95% correct overall. Hence, these children showed comprehension of the ordering of informational strength. Of course, this does not prove that children can derive SIs easily or independently, but it shows their sensitivity to the informational strength of the competing utterances and the importance of the cognitive availability of alternatives.

In Experiment 1, with 5-year-old children as participants, we build on this research by introducing – in addition to choices between “*some*” and “*all*” – also choices between “*some*” and “*many*” and between “*many*” and “*all*,” which makes a more fine-grained analysis possible. In Experiment 2, we present the same problems, but to older children, that is, 11-year-old children, to test developmental patterns. Moreover, we added temporal scales (with “*sometimes*,” “*often*,” and “*always*”) to test scalar diversity.

## Experiment 1: Five-Year-Old Children and Quantitative Scalar Implicatures in a Felicity Judgment Task

As a starting point, Experiment 1 uses the FJT by Foppolo et al. (2012), in which statements with “*some*” and “*all*” were compared as alternative descriptions of pictures in which the statement with “*all*” was the most appropriate. We asked, however, a finer-grained research question: How determining is the generation of alternatives, compared to the evaluation of the information strength itself? In order to have part of the answer to this question, we broadened the FJT of Foppolo et al. (2012). In addition to choices between “*some*” and “*all*,” we also presented choices between “*some*” and “*many*” and “*many*” and “*all*,” and this in situations where “*all*,” “*many*” or “*some*” was the most appropriate according to our intuition. Pezzelle et al. (2018) showed that, for sets with four or more objects, quantifiers primarily represent proportions and not absolute cardinalities. Additionally, even without relying on any quantitative or contextual information, quantifiers lie on an ordered scale, that is, “*none, almost none, few, the smaller part, some, many, most, almost all, all.*” Consequently, in our study “*some*” should be proportionally less than “*many.*”

Table 1 gives an overview of the different types of items. The three possible pairs constructed with “*some*,” “*many*,” and “*all*” were all confronted with situations with two, five, and six successes out of six. For instance, there was a boy throwing rings around the trunk of an elephant. He had six attempts and he succeeded in two (≈ “*some*”), five (≈ “*many*”) or six (≈ “*all*”) attempts. This leads to nine combinations. These combinations can be divided in three categories.

**Table 1:** The nine different items in our adapted Felicity Judgment Task.

*The presented scalar-pairs*	*The type of item^∗^*		
	Critical	Ambiguous	Control
Some-All	SA6	SA5	SA2
Some-Many	SM5	SM6	SM2
Many-All	MA6	MA2	MA5

*^∗^For each item a situation was presented in which six actions were taken, of which six, five or two were successful. The number refers to the number of successes of the main character. Hence, Some-All 6 successes (SA6); Some-All 5 successes (SA5); Some-All 2 successes (SA2); Some-Many 6 successes (SM6); Some-Many 5 successes (SM5); Some-Many 2 successes (SM2); Many-All 6 successes (MA6); Many-All 5 successes (MA5); Many-All 2 successes (MA2).*

The first category consists of three control items (SA2, SM2, MA5), which test the knowledge of the terms, by presenting a pair of assertions, from which one is false and one correct. For instance for item SA2, when there are two successes, the children have to choose between “*some marbles landed in the whole*” and “*all marbles landed in the whole.*” We expect children to perform well on these items, because we expect these items to test the basic lexical/semantic knowledge of the terms used.

The second category consists of the three more or less typical critical items (SA6, SM5, MA6), where an underinformative assertion (“*some*” or “*many*”) is paired with a strong true alternative (“*many*” or “*all*”). For instance for item SA6, when there are six successes, the participants have to choose between “*some arrows landed in the rose*” and “*all arrows landed in the rose.*” If the difficulty of SIs really lies in the generation of alternatives and not in the evaluation of the informational strength, then these items should be answered well. However, given the absence of a comparison process for the control items and a potentially still fragile evaluation system, performance might be lower for the critical items than for the control items.

Finally, the third category contains three ambiguous situations (SA5, SM6, MA2), where none of the alternatives gives a very appropriate description. In item SA5, an underinformative assertion is paired with an assertion that is too strong: in the case of five successes, the underinformative “*some*” is paired with the too strong “*all.*” Consequently, the underinformative “*some*” is the most appropriate choice. In item SM6, two underinformative assertions are paired: in the case of six successes, the underinformative “*some*” is paired with the underinformative “*many.*” Although both assertions are underinformative, one can still make a distinction between them: the difference in informational strength with respect to the six successes (≈ “*all*”) is the smallest with “*many*,” which is therefore the most appropriate choice. In item MA2, two too strong assertions are presented: in the case of two successes, “*many*” is paired with “*all.*” Although both assertions are too strong, the difference in informational strength with respect to the correct two successes (≈ “*some*”) is the smallest with “*many*,” which is therefore the most appropriate choice. Hence, these ambiguous items can be solved only if one is able to compare in a more finely grained fashion the informational structure. Given a potentially still fragile evaluation system, performance is expected to be lower than for the control items and maybe even lower than for the critical items, because no clear right answer was presented.

In sum, in the current FJT we wanted to investigate if 5-year-old children can select the most appropriate term when presented with a choice. On the basis of the literature on the importance of alternatives and on the basis of the work of Foppolo et al. (2012), we expected the children to perform well. We broadened the task, by using also the term “*many.*” We expected on the basis of this broadening that the difficulty of the task would increase. Moreover, the work on alternatives shows that the mere presence of alternatives is not a wonder solution. Consequently, we expected the control items (SA2, SM2, MA5) to be easier than the critical items (SA6, SM5, MA6) and the ambiguous items (SA5, SM6, MA2).

### Methods

#### Participants

We tested 59 5-year-old children (27 boys and 32 girls; mean age = 61 months, *SD* = 3 months). They were all recruited from two primary schools in Belgium. All were native Dutch speakers, including some bilingual children. This research has been reviewed and approved by the ethical review board SMEC of the University of Leuven. A written informed consent was obtained from the participants’ parents.

#### Materials and Procedure

We tested children with a version of the FJT in which we presented two statements, which contained either “*some*” or “*many*” or “*all*” (“*sommige*,” “*vele*,” “*alle*” in Dutch, the language of the experiment; see Appendix A for the material) as alternative descriptions. These statements were accompanied by drawings in which two, five, or six successes were achieved. The children had to decide which statement did fit the drawing best. The children received in total nine stories in a random order.

The participants were tested individually in a quiet space. At the beginning of the experiment, they were told that the investigator would tell a few stories, which she would illustrate with drawings. Next, two animals were introduced, Kwaak the frog and Botje the fish. These two plush hugs were presented to the children as good friends of the researcher. They would both make a statement about each of the stories. It was the child’s task to judge each time which puppet said it better ( = Felicity Judgment Task). Moreover, we took care to assert that there was not one puppet that was always uttering the best statements. Before the experiment started, two practice items were given to familiarize the children with the procedure (see Appendix A).

**FIGURE 1:**
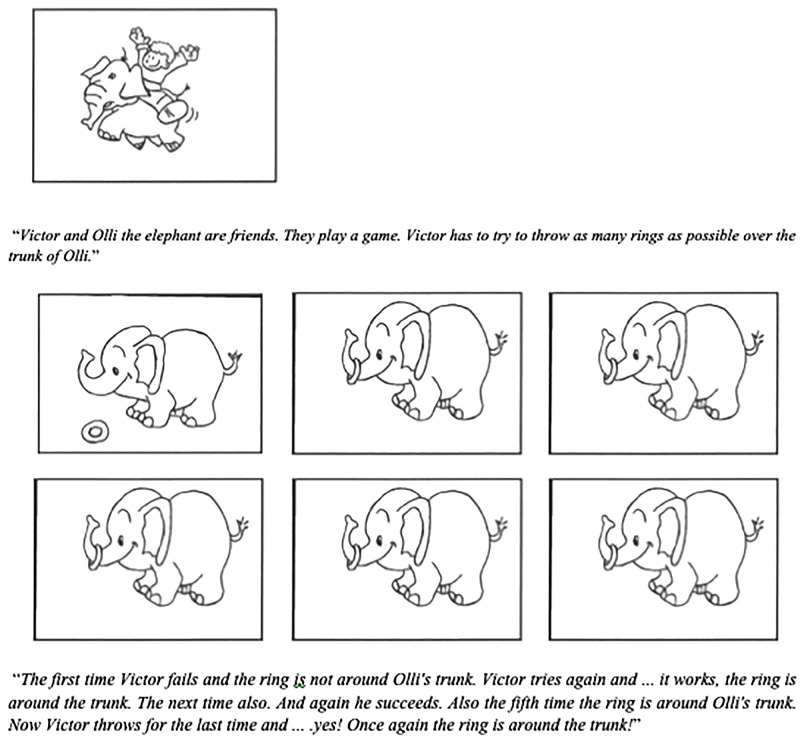
Illustration of one item (with five successes) with the accompanying pictures.

Each experimental item started with a story that was told and which was illustrated by means of drawings, as illustrated in Figure 1. First, the context of the story is told and shown with the contextual drawing. Next it is told how the situation unfolds while six action drawings are shown. For instance, it was told that Victor, a small boy, and Olli, the elephant, are good friends, while a drawing is shown of the two together. Then it is told that they play a game. Victor has to throw six rings around Olli’s trunk. Next, each attempt (success or failure) is described and illustrated with a drawing. For instance, “*The first time Victor fails and the ring is not around Olli’s trunk. Victor tries again and... it works, the ring is around the trunk. The next time also. And again he succeeds. Also the fifth time is the ring around Olli’s trunk. Now Victor throws for the last time and... yes! Once again the ring is sitting around the trunk!*” After this story, both Kwaak and Botje make a statement about the story, and the participant has to indicate which puppet said it better. With the above story, the two statements might be (with between square brackets the English translation):

Kwaak: “*Victor gooide sommige ringen rond Olli’s slurf.*”
[Victor threw some rings around Olli’s trunk]
Botje: “*Victor gooide vele ringen rond Olli’s slurf.*”
[Victor threw many rings around Olli’s trunk]

## RESULTS

Table 2 presents the percentage of appropriate choices for the nine experimental items and Figure 3 depicts the results graphically (together with part of the data of Experiment 2). There was no difference in performance between different versions presented. Overall, the children’s performance in this FJT was quite good, with 87% correct overall and with at least 70% correct. In other words, the 5-year-old children were able to choose clearly above chance which element of the scale < *some, many, all* > from a pair is more appropriate in a given context (Binomial probability = 0.001 for the lowest score, i.e., 70%). Moreover, it is not only that the children, as a group, are better than chance. Only one child scored less than chance level, an additional two children answered less than 2/3 of the problems correctly (but were above ½) and three children precisely answered 2/3 of the problems correctly. In other words, 90% of the children answered more than 2/3 of the problems correctly. Even if we look at the problem types separately, a similar picture emerges. On the critical and ambiguous problems, four children scored less than chance level (these were different children for the critical and ambiguous problems), respectively 19 and 20 children answered 2/3 of the problems correctly and respectively 36 and 35 children answered all three problems correctly. For the control items, two children scored less than chance level, nine children answered 2/3 of the problems correctly and 48 children answered all three problems correctly.

**Table 2:** The proportion of appropriate answers and the standard deviation in Experiment 1.

The presented scalar-pairs	The type of item		
	Critical	Ambiguous	Control
Some-All	0.95 [0.222] (SA6)	0.70 [0.464] (SA5)	0.97 [0.183] (SA2)
Some-Many	0.73 [0.448] (SM5)	0.93 [0.254] (SM6)	0.88 [0.326] (SM2)
Many-All	0.86 [0.345] (MA6)	0.88 [0.326] (MA2)	0.93 [0.254] (MA5)

Given the binary nature of the dependent variable, we performed a mixed effects logistic regression (Baayen et al., 2008; Jaeger, 2008; Bates et al., 2015). The model fitting procedure was implemented in R using the glmer() function from the lme4 package (Bates et al., 2015). The dependent variable was the appropriateness score (1 for appropriate and 0 for inappropriate). The independent variables were Type (with the levels Control, Critical, and Ambiguous) and Quantifier-Pair (with the levels Some-All, Many-All, Some-Many). All models included random intercepts for participants and following Baayen et al. (2008) we additionally opted for a random interaction between Type and participant identifier. We started with the most complex fixed effects structure, including the two-way interaction between Type and Quantifier-Pair and main effects. We conducted likelihood ratio tests (*α* = 0.05) with the mixed function from the afex package to determine the strongest model (Singmann et al., 2018). The model with the interaction was significantly better than the others [χ^2^(4) = 28.23*, p* < 0.00001]. For a complete description of the final model, see Table 3. The control items were significantly easier than the critical items (85% vs. 93%; *Z* = 2.34, *p* = 0.0497) and the ambiguous items (84% vs. 93%; *Z* = 2.18, *p* = 0.0292). We analyzed the significant interaction further by pairwise contrasts, using Bonferroni corrected lsmeans (). This revealed three significant differences for the interaction between Type and Quantifier-pair. For the SA-pairs, the ambiguous item (SA5) was more difficult than the critical item (SA6; 70% vs. 95%; *Z* = 3.36, *p* = 0.0024) and the control item (SA2; 70% vs. 97%; *Z* = −3.42, *p* = 0.0019). For the SM-pairs, the critical item (SM5) was more difficult than the ambiguous item (SM6; 73% vs. 93%; *Z* = −2.50, *p* = 0.0375).

**Table 3:** A complete description of the final model for Experiment 1: Type ^∗^Quantifier-Pair + (1| Participant) + (1| Type: Participant).

Estimators of the relative quality of the statistical model: statistical model:							

AIC	BIC	logLik	deviance	df.resid			
391.2	438.2	−184.6	369.2	520			

***Scaled residuals:***							

**Min**	**1Q**		**Median**		**3Q**	**Max**

−4.8245	0.1746		0.2574		0.3483	1.0312

***Random effects:***						

**Groups**	**Name**		**Variance**	**Standard Deviation**		

Type: Participant	(Intercept)		0.3536	0.5946		
Participant	(Intercept)		0.4528	0.6729		
Number of obs: 531, Type: Participant, 177; Participant, 59							

***Fixed effects°:***							

		**Estimate**	**Standard Error**	***z*-value**	**Pr( > | z| )**	

(Intercept)		2.42135	0.27328	8.860	<2e-16^∗^	
Many-All		0.23252	0.25152	0.924	0.3552	
Some-Many		0.04477	0.22243	0.201	0.8405	
Critical Items		0.57824	0.26523	2.180	0.0292^∗^	
Ambiguous Items		−0.23347	0.23457	−0.995	0.3196	
Many-All: Critical		0.48963	0.39929	1.226	0.2201	
Some-Many Ambiguous guous		0.07821	0.34245	−0.228	0.8194	
Many-All: Critical		0.84582	0.35910	2.355	0.0185^∗^	
Some-Many Ambiguous		−0.10110	0.29951	−0.338	0.7357	

*°The fixed effects are Quantifier-Pair (with the levels Some-All, Many-All, and Some-Many) and Type (with the levels Control, Critical, and Ambiguous).*

### Discussion Experiment 1

We tested if adding an extra term, that is, “*many,”* would lead to similar results as the Foppolo et al. (2012) study. Despite this extra term, the children’s performance in our Felicity Judgment Task was still convincingly above chance level, with all pairs answered appropriately above 70% and with an overall score of 87%. Although these results are in general less good than the ones of Foppolo et al. (2012), where an overall rate of 95% was observed, our results still show that children are able to choose which element of the scale < *some, many, all* > is more appropriate in a given context. In other words, when they are offered with an alternative, they can more or less easily decide which one fits the situation best.

Nevertheless, some interesting differences were observed. As predicted, the critical items were more difficult than the control items. For the latter, the lexical/semantic knowledge does not leave room for doubt about which is the most appropriate answer. For the critical items, the informational strength of the two alternatives has to be compared, in order to provide the correct answer. The necessity of the comparison process for the critical items seems to have caused the lower performance on the critical items. Compared to the control items, performance was also lower for the ambiguous items, which can only be solved by a more sophisticated comparison process: neither of the alternatives is perfect, so a fine-grained comparison is needed. Interestingly, we did not observe a significant difference between the critical and the ambiguous items. In other words, the informational strength evaluation process was sophisticated enough to handle both kinds of items.

The two most difficult items were the ambiguous SA5 and the critical SM5 item. In the ambiguous SA5 item, an underinformative assertion is paired with an assertion that is too strong. Therefore, this item is somewhat different from the two other ambiguous items, where the alternatives are either both too strong or both too weak in terms of informational strength. For the latter two items, one only has to take the distance from the “correct” answer to make the decision. This strategy does not work for the SA5 item, because it leads to the inappropriate (and false) “*all*” choice. “*All*” is indeed in terms of distance closer to five than “*some.*” In other words, it makes sense that this item is more difficult than other items: rather sophisticated inferencing is needed to produce the appropriate answer. Another reason why this item might me more difficult is that “*some*” not only leads to the implicature “*not all*,” but also to the implicature “*not many*,” which then blocks the children. However, the derivation of the “*not many*” implicature by children is unlikely given what we know of their ability to derive SIs. If they would do it anyway, there is a good chance they would see the violation of the implicature as less problematic than the falsity of “*all.*” Why the critical SM5 is also more difficult than other items is less clear. A difference between SM5 and the two other critical items, that is SA6 and MA6, is that the latter two are connected to the endpoint, that is, to the strongest case (six successes). SM5 however is linked to five successes, which is at the top of the scale, but is not an endpoint. This might cause some extra insecurity and therefore explains the lower appropriateness-scores for this item. Support for this hypothesis comes from the work of Van Tiel et al. (2016). They observed for adults large differences between rates of scalar inferences on different scales (between 4 and 100%). One important factor causing these differences was the openness/closeness of the scales. Closed scales (like e.g., <*some, all*>, where “*all*” is the end point) lead to more scalar inferences than open scales (like e.g., <*cool, cold*>, where “*cold*” is not an end point). Unlike < *some, all* >, <*some, many*> is an open scale and maybe therefore more difficult.

## Experiment 2: Eleven-Year-Old Children and Quantitative and Temporal Scalar Implicatures in a Felicity Judgment Task

Although performance was already high, for some items there was clearly room for improvement. In Experiment 2, we investigated whether 11-year-old children would perform better than the 5-year-old children. With respect to the more traditional TVJT, there is a clear developmental trend observed in the literature (see e.g., Pouscoulous et al., 2007). Therefore, we also expected a better performance by the 11-year-old children on our FJT.

Additionally, we wanted to gather some extra data with respect to the issue of scalar diversity. Until recently, the uniformity of SIs had not been questioned. Doran et al. (2009) tested this assumption by looking not only to the scale < some, all> but also to scales like <*possibly, definitely*>, <*beginner, intermediate, advanced*> and <*warm, hot*>. They observed in adults a significant variability between the rates of pragmatic answers that these scalar terms elicit. Likewise, a survey of ten experiments by Geurts (2010, pp. 98–99) showed that, for disjunction sentences (containing “*or*”), the mean rate of SIs was much lower than for the sentences containing “*some*”: 35% against 56.5%. Van Tiel et al. (2016) build further on the work by Doran et al. (2009). Apart from the effect of closed versus open scales, they observed that giving the adjectives a richer context leads to more scalar inferences. Also, word class and semantic distance had a significant effect on the rate of pragmatic responses, while there was no effect of focus, word frequency, or strength of association between stronger and weaker terms. In other words, different types of scales are not all the same and we cannot use one type as the prototypical type. The <*some, all*> scale triggers unusually high levels of pragmatic answers. It is worth noting that recently Benz et al. (2018) provided some support for a modified version of the uniformity hypothesis on the basis of their work on negative strengthening.

To the best of our knowledge, there is no research on scalar diversity with the FJT. Chierchia et al. (2001) already showed good performance with the scale < *or, and*>, Foppolo et al. (2012) with <*some, all*>, but the two scales were not compared. In the current experiment, we directly compared performance on the quantitative scale < *some, many, all* > with the temporal scale^2^ < *sometimes, often, always*>. We opted for these two scales for two reasons. First, they allowed us to use the same materials and procedure. Second, we wanted a scale which was not too difficult for children and Van Tiel et al. (2016) observed for these two scales in adults a high performance. Given the high accuracy of the 5-years-old children in Experiment 1 on the quantitative SIs, we expected not too many difficulties with the temporal SIs.

### Methods

#### Participants

We tested 34 11-year-olds (15 boys and 19 girls; mean age = 11 years; 4 months, *SD* = 5 months). They were all recruited from two primary schools in Belgium. All were native Dutch speakers, including a few bilingual children. A written and informed consent was obtained from the participants’ parents.

#### Materials and Procedure

The same materials and procedure were used as in Experiment 1. The only difference was that the participants had to solve both the Quantitative Scale (QS, as in Experiment 1, with “*all,”* “*many,”* and “*some”*) and the Temporal Scale (TS, with “*always,”* “*often,”* “*sometimes”*). For the exploration of the temporal implicatures the statements that were presented after the context story were rephrased. Consider the example we used in Experiment 1. The same drawings (see Figure 1) were used. First, the same context story was given (Victor, a small boy, and Olli, the elephant, are good friends). Next the ring-throwing game was introduced, with the same sentences and drawings, “*The first time Victor fails and the ring is not around Olli’s trunk. Victor tries again and... it works, the ring is around the trunk. The next time also. And again he succeeds. Also the fifth time is the ring around Olli’s trunk. Now Victor throws for the last time and....yes! Once again the ring is sitting around the trunk!*” After the story, the two puppets made a statement about the story:

Kwaak: “Victor heeft *soms* de ring rond Olli’s slurf geworpen.”
[Victor has *sometimes* the ring around Ollie’s trunk thrown]
Botje: “Victor heeft *altijd* de ring rond Olli’s slurf geworpen.”
[Victor has *always* the ring around Olli’s trunk thrown]

Eighteen participants started with the Quantitative Scale and received afterward the Temporal Scale, while 16 participants started with the Temporal scale. To make the comparison easier, we will use the label SA for both the *some-all* (preceded by Q_) and the *sometimes-always* pairs (preceded by T_), SM for both the *some-many* (preceded by Q_) and the *sometimes-often* pairs (preceded by T_), and MA for both the *many-all* (preceded by Q_) and the *often-always* pairs (preceded by T_).

### Results Experiment 2

**FIGURE 2:**
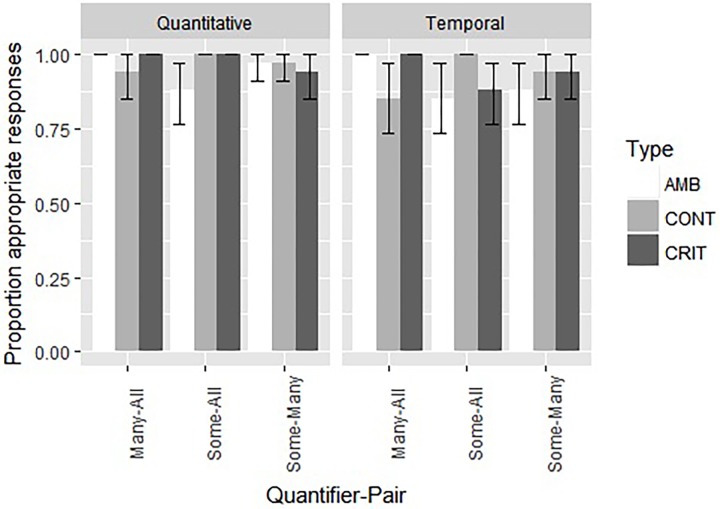
The proportion of appropriate choices and the standard error for both the nine quantitative and the nine temporal items in Experiment 2.

We observed no difference in measurements between the two blocks (starting with the quantitative items vs. starting with the temporal items). Therefore, we collapsed the data over the two orders. Likewise, there was, as in Experiment 1, no difference in performance between the different versions presented. Table 4 presents the percentage of appropriate choices for the nine experimental items for the two scales and Figure 2 depicts the results graphically.

**Table 4:** The proportion of appropriate answers and the standard deviation in Experiment 2.

The presented scalar-pairs	The type of item		
	Critical	Ambiguous	Control
Q_Some-All^∗^	100 [0.000]	88 [0.327]	100 [0.000]
Q_Some-Many	94 [0.239]	97 [0.172]	97 [0.172]
Q_Many-All	100 [0.000]	100 [0.000]	94 [0.239]
T_Some-All	88 [0.327]	85 [0.360]	100 [0.000]
T_Some-Many	94 [0.239]	88 [0.327]	94 [0.239]
T_Many-All	100 [0.000]	100 [0.000]	85 [0.360]

*^∗^Q_ indicates the quantitative items, T_ indicates the temporal items.*

Overall, performance on the Quantitative items in this FJT was very good, with 97% correct overall and with at least 88% correct (Binomial probability = 0.001). For the Temporal items, a similar pattern was observed: performance was very good, with 93% correct overall and with at least 85% correct (Binomial probability = 0.001). All children answered more than 2/3 of the quantitative items correctly; 33 children answered more than 2/3 of the temporal items correctly, two children answered precisely 2/3 of the temporal items correctly. If we look at the problem types separately, a similar picture emerges. On the quantitative and temporal critical items, respectively two and six children answered 2/3 of the problems correctly, and respectively 33 and 29 children answered all three problems correctly. On the quantitative and temporal ambiguous items, respectively five and seven children answered 2/3 of the problems correctly, and respectively 30 and 28 children answered all three problems correctly. On the quantitative and temporal control items, three children answered 2/3 of the problems correctly, three children answered only 1 of the temporal items correctly, and respectively 32 and 29 children answered all three problems correctly.

As for Experiment 1, we performed a mixed effects logistic regression, with the model fitting procedure glmer() function from the lme4 package (Bates et al., 2015). The dependent variable was the appropriateness score (1 for appropriate and 0 for inappropriate). The independent variables were Type (with the levels Control, Critical, and Ambiguous), Quantifier-Pair (with the levels Some-All, Many-All, and Some-Many), and Diversity (Quantitative and Temporal). All models included random intercepts for participants. This model and other more complex models failed to converge, possibly due to ceiling effects. Non-parametric analyses confirm a lack of differences between the different conditions and the very high performance (see Appendix B). However, of the simple models, the one with Diversity as a factor was the best [model fitting verified through the Akaike information criterion (AIC) and the BIC]. The estimation of the fixed effects of Diversity was consistent, even if we had a more complex random structure, for instance by including a random interaction between participants and Type. However, we opted for the model without problems with convergence and degenerated random effects, which is the one with only a random intercept for participants. This model indicates that the temporal items were more difficult than the quantitative ones (93% vs. 97%; *Z* = 2.20, *p* = 0.0278), a difference also confirmed through non-parametric analyses. For a complete description of this simple final model, see Table 5.

**Table 5:** A complete description of the simple model for Experiment 2: Diversity + (1| Participant).

Estimators of the relative quality of the statistical model: statistical model:							

AIC	BIC	logLik	deviance	df.resid			
240.9	254.2	−117.5	234.9	609			

*Scaled residuals:*						

**Min**	**1Q**		**Median**		**3Q**	**Max**

−4.5978	0.1051		0.1638		0.2175	0.6007

*Random effects:*						

**Groups**	**Name**		**Variance**	**Standard. deviation**		

Participant	(Intercept)		1.457	1.207		

Number of obs: 612, Participant: 34						

***Fixed effects°:***						

		**Estimate**	**Standard Error**	***z* value**	**Pr( > | z| )**	

(Intercept)		4.0209	0.4940	8.139	3.98e-16^∗^	
Diversity		−0.8880	0.4036	−2.200	0.0278^∗^	

*°The fixed effect is Diversity (with the levels Quantitative and Temporal).*

### Comparison Results Experiments 1 and 2

**FIGURE 3:**
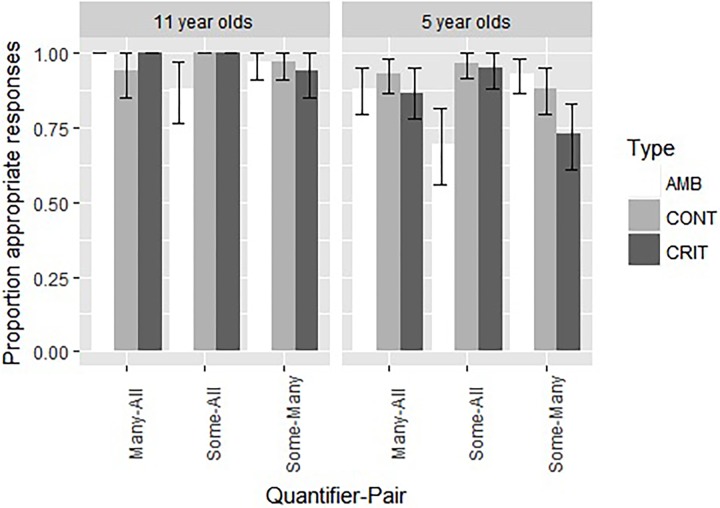
A comparison of the proportion of appropriate choices and the standard error for the nine quantitative items in Experiment 1 (5-year-old children) and Experiment 2 (11-year-old children).

We also compared the performance on the quantitative items between the younger age group of Experiment 1 and the older group of Experiment 2. These data are presented graphically in Figure 3. As before, we performed a mixed effects logistic regression, with the model fitting procedure glmer() function from the lme4 package (Bates et al., 2015). The dependent variable was the appropriateness score (1 for appropriate and 0 for inappropriate). The independent variables were Type (with the levels Control, Critical, and Ambiguous), Quantifier-Pair (with the levels Some-All, Many-All, and Some-Many), and Age (5-year-olds vs. 11-years-old). As for Experiment 1, all models included random intercepts for participants and a random interaction between Type and participant identifier. We started with the most complex fixed effects structure, including the three-way interaction, two-way interactions and main effects. We conducted likelihood ratio tests (*α* = 0.05) with the mixed function from the afex package to determine the strongest model. The best model contained Age (χ*2*(1) = 23.32*, p < .*00001) and the interaction between Quantifier-Pair and Type (χ*2*(4) = 25.23, *p* < 0.00001). For a complete description of the final model, see Table 6. The 11-year-olds performed significantly better than the 5-year-olds (97% vs. 85%; *Z* = 4.39, *p* < 0.00001). We analyzed the significant interaction further by pairwise contrasts, using Bonferroni corrected lsmeans (). This revealed three significant differences for the interaction between Type and Quantifier-pair. For the SA-pairs, the ambiguous item (SA5) was more difficult than the critical item (SA6; 79% vs. 98%; *Z* = 3.36, *p* = 0.0024) and the control item (SA2; 79% vs. 97%; *Z* = −3.42, *p* = 0.0019). For the SM-pairs, the critical item (SM5) was more difficult than the ambiguous item (SM6; 85% vs. 95%; *Z* = −2.50, *p* = 0.0375).

**Table 6:** A complete description of the final model for Experiment 1: Age + Type ^∗^Quantifier-Pair + (1| Participant) + (1| Type: Participant).

Estimators of the relative quality of the statistical model:							

AIC	BIC	logLik	deviance	df.resid			
472.9	529.7	−224.5	448.9	825		

***Scaled residuals:***						

**Min**	**1Q**		**Median**		**3Q**	**Max**

−7.7700	0.1206		0.2103		0.3084	0.9694

***Random effects:***						

**Groups**	**Name**		**Variance**	**Standard Deviation.**		

Type: Participant	(Intercept)		0.00533	0.07303		
Participant	(Intercept)		0.529134	0.72742		
Number of obs: 837, Type: Participant, 177; Participant, 59							

***Fixed effects°:***						

		**Estimate**	**Standard Error**	***z* value**	**Pr( > | z| )**	

(Intercept)		2.19039	0.42243	5.185	2.16e-07^∗^	
Age 11		1.61636	0.37028	4.365	1.27e-05^∗^	
Some-All		1.09034	0.70911	1.538	0.1241	
Some-Many		−1.04144	0.47935	−2.173	0.0298^∗^	
Ambiguous		0.15599	0.55943	0.279	0.7804	
Control		0.33225	0.58069	0.572	0.5672	
Some-All: Ambiguous		−2.58529	0.86495	−2.989	0.0028^∗^	
Some-Many Ambiguous guous		1.42184	0.78602	1.809	0.0705	
Some-All: Control		0.09507	1.10093	0.086	0.9312	
Some-Many: Control		0.70954	0.75133	0.944	0.3450	

*°The fixed effects are Age (with the levels Age_5 and Age_11), Quantifier-Pair (with the levels Many-All, Some-All, and Some-Many), and Type (with the Critical, Ambiguous, and Control).*

## General Discussion

In two experiments, we tested the ability of both 5-year-old and 11-year-old children to select the most appropriate item in a FJT. The set-up of our experiments was inspired by Foppolo et al. (2012), but we broadened the typical <*some, all*> scale to a <*some, many, all*> scale. Two aspects seem immediately relevant.

First, both age groups performed well above chance level. When asked to choose which of the two alternatives is the best description, the children were good in making the right decision. In other words, with the alternatives explicitly presented, even young children are able to pick the pragmatically most appropriate option. Second, despite performing at a high level, the 5-year-old children were less able to choose the appropriate answer compared to the 11-year-old children. Interestingly, this difference was not observed on the control items, but only on the critical and on the ambiguous items. Likewise, for the group of 5-year-old children separately, the critical and the ambiguous items were more difficult than the control items.

These findings are important for the literature about the role of alternatives. Our data confirm the claim that the explicit presence of alternatives eases pragmatic reasoning for young children (see e.g., Barner et al., 2011). Young children seem to be able to pick the most appropriate answer, which is the only correct one in the case of the control items and the one with the most information strength in the case of the critical and most ambiguous items. However, our data also point to the importance of the comparison process. The critical and the ambiguous items were more difficult than the control items. So, the mere presence of the most appropriate alternative is not enough to elicit performance at ceiling level. For the critical and the ambiguous items, the information strength of the two alternatives has to be compared and this seems to have increased the difficulty level. We have to emphasize that even for these items the performance was clearly above chance level: children can reliably solve these problems. However, performance was lower on these items than on the control items, which can be interpreted as a sign of the processing load of the comparison process or of the intrinsic difficulty of the comparison itself. This interpretation is in line with the constraint-based approach of SIs (Degen and Tanenhaus, 2015, 2016), which claims that the probabilistic support for the implicature in context determines the probability of a SI and the speed at which it is derived (see e.g., Breheny et al., 2006 for earlier results in this direction). Greater contextual support leads to a higher probability for the implicature and a faster derivation. The explicit use of a third alternative in the experiments (not only “*some”* and “*all,”* but also “*many”*) could have complicated the process. It is indeed conceivable that, in contrast to the Foppolo et al. (2012) study, the children in the current study spontaneously assumed that a bigger set of alternatives was available for the speaker, which in turn affected the difficulty of the inferences drawn. Degen and Tanenhaus (2016) for instance showed that the availability of lexical alternatives outside the <*all-some*> scale, that is, number alternatives, increased the difficulty of interpreting “*some.”* In our experiment, the introduction of “*many”* might have played a similar role. It is possible that this was especially the case for the youngest children. Moreover, the observed difficulty with the critical and the ambiguous items is in agreement with the idea that the contextual support for their appropriate choices is less strong than for the appropriate choices for the control items.

Given that especially two items (i.e., SA5 and SM5) were more difficult for the youngest children, we believe that it’s not so much the general processing load of the comparison process itself which caused the effect, but the intrinsic difficulty of some comparisons. For both items, it can be argued that the most appropriate choice received less contextual support compared to the other items. In hindsight, it is therefore not surprising that the ambiguous SA5 and the critical SM5 turned out to be the hardest ones. The ambiguous SA5 item is the only ambiguous item where an underinformative assertion is paired with a too strong assertion. For this item, the child had to realize that the shorter distance between “*all*” (i.e., six successes) and five successes, compared to the distance between “*some*” (i.e., at least one success) and the five successes, has to be neglected, given the fact that “*all*” is too strong in this case. For the other two ambiguous items, the alternatives were either both too strong or both too weak in terms of informational strength, which enabled the children to focus only on the distance from the “correct” answer for their decision. In the discussion of Experiment 1, we mentioned another potential explanation for the difficulty of SA5. “*Some*” might not only elicit a “*not all*” implicature, but also a “*not many*” implicature, which consequently might have blocked the children. However, this clearly is a rather sophisticated inferencing, which you would not expect from the youngest children, but maybe from the older ones. Given the fact that the 11-year-old did not struggle so much with this item, we believe that this explanation is unlikely, although it cannot be completely ruled out on the basis of our study. Future research should look further into this issue. A related factor is the potential effect of order of presentation, which might definitely be of importance for the ambiguous and critical items. As written in the results sections, in our experiments there was no order effect. However, we only presented nine different items and we did therefore not present similar items after each other. Suppose participants receive a few ambiguous items of the SA5-type. This item forces them to accept “*some*” with five successes (or “*all*” with five successes). Multiple presentations of this item might consequently have an influence on the subsequent items with “*some.*” Using reaction times as an extra dependent variable is clearly advisable here. The critical SM5 item is also special, because the endpoint, that is, the strongest case (six successes), is not part of the comparison process. Van Tiel et al. (2016) already observed for adults that scales with an endpoint lead to more scalar inferences than scales without.

Experiment 2 showed that with development, children are able to deal with these more difficult items. For the 11-year-old children, there was no difference between the control, critical, and ambiguous quantitative items, and also pairwise comparisons between the nine different items revealed no significant differences. In other words, at that age, when presented with two alternatives, irrespective of the difficulty of the comparison process, the 11-year-old children are able to pick the most appropriate quantitative description. This is maybe not very surprising because at age eleven children seem to be able to perform a large range of pragmatic inferences (but not all, see e.g., Janssens et al., 2015 on conventional implicatures). For instance, the age of ten is critical for metaphor (Lecce et al., 2018), idiom (Kempler et al., 1999), and irony understanding (Glenwright and Pexman, 2010). It will be interesting to see in future research how children younger than five behave on the current task: Which items will be the most difficult for them and from which age is performance above chance level? We know that the classic TVJT with SIs is often too difficult for 3-year old children (e.g., Hurewitz et al., 2006; Janssens et al., 2014), but with contextually grounded, *ad hoc* implicatures children by age three and a half, and perhaps even slightly earlier, can cope with it (see e.g., Stiller et al., 2015). Similarly, Tieu et al. (2016) showed that 4-year-old children could compute free choice inferences but not SIs. Given the high performance on our task, we can expect already above chance performance for the 3.5 year old children. Future research could also investigate how performance is with other numbers. Here we opted for a maximum of six potential successes, given the young age of our participants in Experiment 1. Not only will it be interesting to see how children cope with situations with a higher number of potential successes, this manipulation would also give the opportunity to play a bit more with the set-sizes attached to “*some*” and “*many.*” Additionally, such a manipulation would provide evidence about which conditions trigger which quantifiers easier, because it is perfectly conceivable that some set-sizes are better fits for “*some*” or “*many*” than others (see also Degen and Tanenhaus, 2015). There is some work on this with adults (see e.g., Newstead and Coventry, 2000; Coventry et al., 2005, 2010; Van Tiel, 2014; Pezzelle et al., 2018), but to the best of our knowledge not with children. Especially relevant for our results might be the observation of Pezzelle et al. (2018) that both low- and high-magnitude quantifiers are ordered along a scale, but that the high-magnitude quantifiers are extremely close to each other, which indicates that their representations overlap. This kind of overlap or “confusion” might be bigger for young children, and might explain some of the difficulties that they experience with “*many.*” Finally, manipulating the range of number of items also opens an extra link with the work of Degen and Tanenhaus (2015, 2016), which showed that “*some*” competes with numbers in the subitizing range, which caused a slower processing.

The results of Experiment 2 teach us that, although we explicitly opted for very similar scales that elicited high numbers of scalar responses from adults (Van Tiel et al., 2016), the temporal items were somewhat more difficult than the quantitative ones for the 11-year-old children. We want to emphasize, however, that the difference is small and only present in a simple model of the data and needs replication in subsequent research. A reason for the observed difference might be found in the stories that we used to introduce each pair of utterances. In these stories, we mentioned a success or a failure, one after the other, and so on, until six events were described. Although this can be seen as a temporal framework, no explicit temporal information was given. The mere mentioning of the different attempts one after the other might have therefore advantaged the quantitative implicatures. If that is the case, we can expect a bigger difference between the two scales for the younger children. This is also of interest for future research. Nowadays there seems to be great concern for the diversity of scalar expressions, with Van Tiel et al. (2016) as a great example (see also e.g., Doran et al., 2009; Geurts, 2010). However, from a developmental point of view, clearly much more research is necessary. Also interesting in this respect is the observation that the most difficult critical item in Experiment 1 was one where the endpoint of the scale was not involved in the comparison process. Van Tiel et al. (2016) already argued that scales with and without an endpoint differ from each other.

A last consideration from our data concerns our use of the extra term *“many.”* This is not the first demonstration that a small change in a simple experiment investigating SIs can lead to important differences in behavioral patterns. The introduction by Katsos and Bishop (2011) of a middle option in the classic binary TVJT (‘I do agree’ vs. ‘I disagree’ became ‘I totally agree,’ ‘I agree a bit,’ and ‘I totally disagree’) proved to be crucial in developmental studies. In the binary task children accept underinformative sentences while adults reject them. When a middle option is present, both adults and children clearly prefer this middle option. Hence, it seems that in the binary task children are not insensitive to underinformativeness, but they do not show it, whereas in the ternary task sensitivity to informativeness is demonstrated through the possibility of showing tolerance to violations of informativeness, by choosing the middle value for underinformative statements. Wampers et al. (2017) and Schaeken et al. (2018) evidenced that, with such a ternary task, respectively patients with psychosis and children with autism spectrum disorder produce less pragmatic responses, while such a difference was not observed with the classic binary task. In other words, a more nuanced task revealed a previously not visible effect, casting new light on the range of pragmatic difficulties in atypical populations. Similarly, in the current study, the introduction of some extra pairs revealed a subtle but important shortcoming in the 5-year-old children, which was absent in the older children and which was not visible in a more simple experiment.

In sum, the current research elucidated the underlying processes connected with scalar alternatives. In a Felicity Judgment Task, where the alternative is given, both the 5- and 11-year-old children performed above chance on all items. However, for the 5-year-old children, the critical and ambiguous items were more difficult than the control items and they also performed worse on these two items than the 11-year-old children. Interestingly with respect to the issue of scalar diversity, the 11-year-old children were also presented temporal items, which turned out to be more difficult than the quantitative ones.

## Ethics Statement

This research has been reviewed and approved by the ethical review board SMEC (Sociaal-Maatschappelijke Ethische Commissie; Social and Societal Ethics Committee) of the University of Leuven. Informed consent was obtained from the participants’ parents in accordance with the Declaration of Helsinki.

## Author Contributions

All authors contributed to this article, both substantially and formally. WS and KD designed the study and interpreted the data. BS prepared the experiment, constructed the stimuli, performed the experiments, and statistical analysis under supervision of WS and KD. BS wrote the first draft of the method and result section. WS wrote the introduction and the general discussion, and revised the methods and results sections. All authors approved the final version of the manuscript.

## Conflict of Interest Statement

The authors declare that the research was conducted in the absence of any commercial or financial relationships that could be construed as a potential conflict of interest.

## Footnotes

1 Two recent studies with adults also highlighted the effect of activating alternatives. In an eye tracking study, Foppolo and Marelli (2017) obtained new evidence for the incremental derivation of the pragmatic some-but-not-all interpretation of “*some.*” They interpret these findings within the grammatical account of SI (e.g., Chierchia et al., 2012): when scalar alternatives are active, the SIs are factored in locally and incrementally during the online processing of scalar quantifiers. With a structural priming paradigm, Rees and Bott (2018) convincingly demonstrated that adults are sensitive to the salience of alternatives when deriving scalar implicatures.

2 In order to avoid confusion, we explicitly mention that we use the term “temporal scalar implicature” differently from other authors, e.g., Altshuler and Schwarzschild (2012) and Thomas (2012), who use it to describe the situation where one infers from “*John was in his office*” that “*John isn’t in his office now/anymore.*”
