# Supplementary material for: Development of Quantitative and Temporal Scalar Implicatures in a Felicity Judgment Task
Source: Front Psychol. 2019 Feb 18;9:2763. doi: 10.3389/fpsyg.2018.02763 (PMC6387925; doi:10.3389/fpsyg.2018.02763)
Supplement: Supplementary file 1 [file Data_Sheet_1.PDF]

Appendix A: An example of the pairs used in Experiment 1 and 2 and the practice items.

Some/sometimes vs. All/Always

Kwaak: “Ruben schiet sommige pijlen raak.”
Kwaak: “Ruben hits with some arrows.”

Kwaak: “Ruben schiet soms de pijl raak.”
Kwaak: “Ruben hits sometimes with the arrow.”

Botje: “Ruben schiet met alle pijlen raak.”
Botje: “Ruben hits with all the arrows.”

Botje: “Ruben schiet altijd de pijl raak.”
Botje: “Ruben hits always with the arrows.”

Kwaak: “Felix vangt sommige visjes.”
Kwaak: “Felix catches some fish.”

Kwaak: “Felix vangt soms het visje.”
Kwaak: “Felix catches sometimes the fish.”

Botje: “Felix vangt alle visjes.”
Botje: “Felix catches all the fish.”

Botje: “Felix vangt altijd de visjes.”
Botje: “Felix catches always the fish.”

Kwaak: “Amber blaast sommige kaarsjes uit.”
Kwaak: “Amber blows some candles out.”

Kwaak: “Amber blaast soms het kaarsje uit.”
Kwaak: “Amber blows sometimes the candle out.”

Botje: “Amber blaast alle kaarsjes uit.”
Botje: “Amber blows all the candles out.”

Botje: “Amber blaast altijd het kaarsje uit.”
Botje: “Amber blows always the candle out.”

Some/sometimes vs. Many/often

Kwaak: “Fleur heeft sommige bloemen zien opkomen ”
Kwaak: “Fleur has some flowers seen emerge.”

Kwaak: “Fleur heeft soms de bloem zien opkomen.”
Kwaak: “Fleur has sometimes the flower seen emerge."

Botje: “Fleur heeft vele bloemen zien opkomen.”
Botje: “Fleur has many flowers seen emerge.”

Botje: “Fleur heeft vaak de bloem zien opkomen.”
Botje: “Fleur has often the flower seen emerge.”

Kwaak: “Valerie heeft sommige lieveheersbeestjes met stippen.”
Kwaak: "Valerie has some ladybugs with dots."

Kwaak: “Valerie heeft soms het lieveheersbeestje met stippen.”
Kwaak: “Valerie has sometimes the ladybugs with dots.”

Botje: “Valerie heeft vele lieveheersbeestjes met stippen.”
Botje: “Valerie has many ladybirds with dots.”

Botje: “Valerie heeft vaak het lieveheersbeestje met stippen.”
Botje: “Valerie has often the ladybird with dots.”

Kwaak: “Victor gooit sommige ringen rond Olli’s slurf.”
Kwaak: “Victor throws some rings around Olli’s trunk.”

Kwaak: “Victor gooit soms de ring rond Olli’s slurf.”
Kwaak: “Victor throws sometimes the rings around Olli’s trunk.”

Botje: “Victor gooit vele ringen rond Olli’s slurf.”
Botje: “Victor throws many rings around Olli’s trunk.”

Botje: “Victor gooit vaak de ring rond Olli’s slurf.”
Botje: “Victor throws often the ring around Olli’s trunk.”

Many/often vs. All/allways

Kwaak: “Maarten rolt vele knikkers in het kuiltje.”
Kwaak: “Maarten rolls many marbles into the dimple.”

Kwaak: “Maarten rolt vaak de knikker in het kuiltje.”
Kwaak: “Maarten rolls often the marble into the dimple."

Botje: “Maarten rolt alle knikkers in het kuiltje.”
Botje: "Maarten rolls all the marbles in the dimple.”

Botje: “Maarten rolt altijd de knikker in het kuiltje.”
Botje: “Maarten rolls always the marble in the dimple.”

Kwaak: “Dries heeft vele eieren gevonden. ”
Kwaak: “Dries has many eggs found.”

Kwaak: “Dries heeft vaak het ei gevonden. ”
Kwaak: “Dries has often the egg found.”

Botje: “Dries heeft alle eieren gevonden.”
Botje: “Dries has all eggs found.”

Botje: “Dries heeft altijd het ei gevonden.”
Botje: “Dries has always the egg found.”

Kwaak: “Stef schopt vele ballen in het doel.”
Kwaak: "Stef kicks many balls into the goal.”

Kwaak: “Stef schopt vaak de bal in het doel.”

Kwaak: “Stef kicks often the ball into the goal.”

Botje: “Stef schopt alle ballen in het doel.”
Botje: “Stef kicks all the balls in the goal.”

Botje: “Stef schopt altijd de bal in het doel.”
Botje: “Stef kicks allways the ball in the goal.”

Practice item 1:

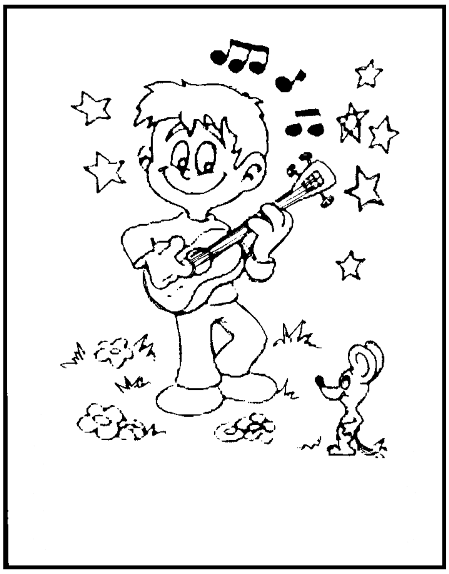

Wouter is a boy who loves music. He can also play music very well! And when he does that, the musical notes dance around and his friends, like the mouse, listen. If you look to the drawing, who is right, Kwaak or Botje?
Kwaak: "Wouter plays the flute."
Botje: "Wouter plays the guitar."

Practice item 2:

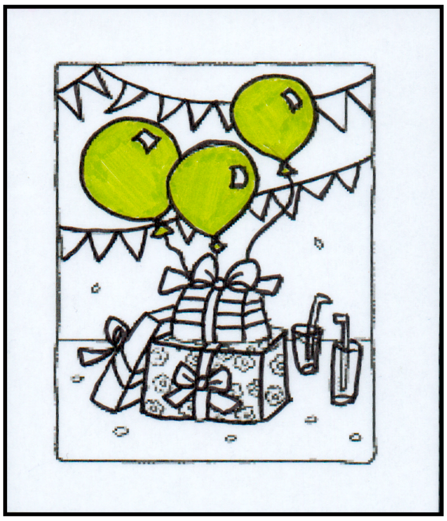

It's party time and like at all parties there are lots of presents, tasty cakes and drinks. There are also garlands and balloons fly through the room. If you look to the drawing, who is right, Kwaak or Botje?

Kwaak: "All balloons have the same color."

Botje: "All balloons have a different color."
