# Supplementary material for: Development of Quantitative and Temporal Scalar Implicatures in a Felicity Judgment Task
Source: Front Psychol. 2019 Feb 18;9:2763. doi: 10.3389/fpsyg.2018.02763 (PMC6387925; doi:10.3389/fpsyg.2018.02763)
Supplement: Supplementary file 2 [file Data_Sheet_2.PDF]

Appendix B: Non-parametric analysis of the experiments

Experiment 1 (by means of Wicoxon's T):
There were no differences between the three pairs. SA (87%), SM (85%) and MA (89%) did not differ significantly from each other. However, we observed a difference with respect to the number of successes. Situations with five successes were more difficult than the ones with six successes (79% vs 92%; Wilcoxon's $T = 341.5$, $p = .005$) and the ones with two successes (79% vs 91%; Wilcoxon's $T = 85$, $p = .001$). To further investigate the difficulty of the specific pairs, we additionally performed pairwise comparisons, whereby we corrected for multiple-testing using Bonferonni correction. This analysis showed that two items turned out to be different from the other items, one ambiguous item (SA5) and one critical item (SM5). The ambiguous item SA5 was significantly more difficult than two control items, that is, SA2 (70% vs. 97%; Wilcoxon's $T = 9.5$, $p = .005$) and MA5 (70% vs. 93%; Wilcoxon's $T = 19$, $p = .001$), than critical item SA6 (70% vs. 95%; Wilcoxon's $T = 20$, $p = .001$) and then ambiguous item SM6 (70% vs. 93%; Wilcoxon's $T = 19$, $p = .001$). For the critical item SM5, exactly the same significant differences were observed; that is, SM5 was significantly more difficult than two control items, that is, SA2 (73% vs. 97%; Wilcoxon's $T = 8.5$, $p = .001$) and MA5 (73% vs. 93%; Wilcoxon's $T = 28.5$, $p = .005$), than critical item SA6 (73% vs. 95%; Wilcoxon's $T = 30$, $p = .003$), and than ambiguous item SM6 (70% vs. 93%; Wilcoxon's $T = 28.5$, $p = .005$).

Experiment 2 (by means of Wicoxon's T):
The control, the critical and the ambiguous quantitative items (97%, 98% and 95% respectively) did not differ significantly from each other. Similarly, Q_SA, Q_SM and Q_MA (96%, 96% and 98% respectively) did not differ significantly from each other. There was also no difference with respect to the number of successes. Situations with six (95%), five (92%) and two successes (99%) did not differ from each other. Moreover, none of the pairwise comparisons between the specific pairs turned out to be (Bonferroni-corrected) significant.

With the temporal items, more or less the same results were observed. Overall, performance was very good again, with 93% correct overall and with at least 85% correct (Binomial probability = .001). The control, the critical and the ambiguous temporal items (94%, 93%, and 91% respectively) did not differ significantly from each other. Similarly, T_SA, T_SM and T_MA (91%, 92% and 95% respectively) did not differ significantly from each other. There was, however, a difference with respect to the number of successes. Situations with two successes were solved better than those with five successes (98% vs. 88%; Wilcoxon's $T = 55$, $p = .0256$). Moreover, none of the pairwise comparisons between the specific pairs turned out to be (Bonferroni-corrected) significant.

When we compare the quantitative and the temporal items, the quantitative items are overall easier than the temporal items (97% vs 93%; Wilcoxon's $T = 10.5$, $p = .024$). None of the other comparisons turned out to be significant.

Comparison of the quantitative items of Experiment 1 and 2 (by means of Mann-Whitney U):
Overall, the five-year-old children performed worse than the eleven-year-old children (87% vs. 97%; Mann-Whitney $U = 526$, $p < .001$). For the control items, there was no significant difference between the two age-groups (92% vs. 99%), but the younger group performed worse on both the critical items (85% vs. 98%; Mann-Whitney $U =$

667, $p < .001$) and on the ambiguous items (84% vs. 95%; Mann-Whitney $U = 732.5$, $p = .008$). SA, SM and MA were all significantly more difficult for the younger age-group than for the older group (respectively, 87% vs. 96%, Mann-Whitney $U = 760$, $p = .012$; 85% vs. 96%, Mann-Whitney $U = 706$, $p = .003$; 89% vs. 98%, Mann-Whitney $U = 771$, $p = .008$). There was no difference on the situations with six successes, but the younger children performed worse on both situations with five successes (79% vs. 92%; Mann-Whitney $U = 650$, $p = .001$) and the ones with two successes (91% vs. 99%; Mann-Whitney $U = 777$, $p < .006$). Comparisons for each of the items separately did not lead to (Bonferroni-corrected) significant differences between the young and old age-groups.
